# Method for Diagnosing the Bone Marrow Edema of Sacroiliac Joint in Patients with Axial Spondyloarthritis Using Magnetic Resonance Image Analysis Based on Deep Learning

**DOI:** 10.3390/diagnostics11071156

**Published:** 2021-06-24

**Authors:** Kang Hee Lee, Sang Tae Choi, Guen Young Lee, You Jung Ha, Sang-Il Choi

**Affiliations:** 1 Department of Computer Science and Engineering, Dankook University, Yongin-si 16890, Korea; hot093054@naver.com; 2 Division of Rheumatology, Department of Internal Medicine, Chung-Ang University College of Medicine, Seoul 06973, Korea; beconst@cau.ac.kr; 3 Department of Radiology, Chung-Ang University College of Medicine, Seoul 06973, Korea; netty0523@cau.ac.kr; 4 Division of Rheumatology, Department of Internal Medicine, Seoul National University Bundang Hospital, Yongin-si 13620, Korea; haayou@hanmail.net; 5 Department of Computer Engineering, Dankook University, Yongin-si 16890, Korea

**Keywords:** axial spondyloarthritis, bone marrow edema, sacroiliitis, magnetic resonance imaging, deep learning

## Abstract

Axial spondyloarthritis (axSpA) is a chronic inflammatory disease of the sacroiliac joints. In this study, we develop a method for detecting bone marrow edema by magnetic resonance (MR) imaging of the sacroiliac joints and a deep-learning network. A total of 815 MR images of the sacroiliac joints were obtained from 60 patients diagnosed with axSpA and 19 healthy subjects. Gadolinium-enhanced fat-suppressed T1-weighted oblique coronal images were used for deep learning. Active sacroiliitis was defined as bone marrow edema, and the following processes were performed: setting the region of interest (ROI) and normalizing it to a size suitable for input to a deep-learning network, determining bone marrow edema using a convolutional-neural-network-based deep-learning network for individual MR images, and determining sacroiliac arthritis in subject examinations based on the classification results of individual MR images. About 70% of the patients and normal subjects were randomly selected for the training dataset, and the remaining 30% formed the test dataset. This process was repeated five times to calculate the average classification rate of the five-fold sets. The gradient-weighted class activation mapping method was used to validate the classification results. In the performance analysis of the ResNet18-based classification network for individual MR images, use of the ROI showed excellent detection performance of bone marrow edema with 93.55 ± 2.19% accuracy, 92.87 ± 1.27% recall, and 94.69 ± 3.03% precision. The overall performance was additionally improved using a median filter to reflect the context information. Finally, active sacroiliitis was diagnosed in individual subjects with 96.06 ± 2.83% accuracy, 100% recall, and 94.84 ± 3.73% precision. This is a pilot study to diagnose bone marrow edema by deep learning based on MR images, and the results suggest that MR analysis using deep learning can be a useful complementary means for clinicians to diagnose bone marrow edema.

## 1. Introduction

Spondyloarthritis (SpA) refers to a set of interrelated rheumatic diseases comprising ankylosing spondylitis, psoriatic arthritis, spondylitis with inflammatory bowel disease, and reactive arthritis [1]. Patients with SpA can be distinguished by their clinical presentation as having predominantly axial SpA (axSpA) or predominantly peripheral SpA [1]. Axial SpA is a chronic inflammatory disease that predominantly presents as inflammation of the sacroiliac joints (SIJs) accompanied by inflammation of the spine and entheses. Axial SpA can be divided into radiographic and non-radiographic axSpA depending on whether definitive structural changes are evident in the SIJs on plain radiographs [2]. Radiographic axSpA indicates an advanced status with bony changes in the SIJs; however, these bony changes are irreversible, so early diagnosis and early treatment of SpA are important [3].

The traditional imaging modality used for examining SIJ lesions is X-ray. However, diagnosis of sacroiliitis from plain radiographs has several critical limitations, the most important being that diagnosis and grading of structural changes in the SIJs show poor reproducibility and inconsistent outcomes among rheumatologists and radiologists [2,4]. Moreover, abnormalities manifest only at advanced stages of disease progression in plain X-ray imaging [5].

Computer tomography (CT) is another common imaging technique to detect structural changes in the SIJs [6]. CT has some advantages over plain radiography in that it permits multiplanar assessments for optimal analysis [7,8]. However, CT use is generally limited because of the risk of higher radiation exposure than plain radiography [9]. More importantly, CT also has limitations for detecting sacroiliitis at the non-radiographic stage, and the agreement between plain radiography and CT data was very poor, with a kappa value of 0.2418 [8,10].

Owing to these limitations, one of the most important methods for early diagnosis of active sacroiliitis in axSpA is via magnetic resonance (MR) imaging [11,12]. In general, sacroiliitis progresses in two stages; inflammation first occurs in the SIJs, and deformation of the joints occur subsequently at the location of inflammation. MR imaging is useful for early diagnosis of non-radiographic axSpA since it can distinguish early inflammation without the obvious structural changes, unlike X-ray and CT, which can only detect structural changes [11,12]. The initial inflammation related to axSpA manifests in the form of bone marrow edema, which is indicated by an increase in the signal intensity in the gadolinium-enhanced fat-suppressed T1-weighted MR image. However, analyses of MR results also have limitations for disease diagnosis because the concordance rate among specialists remains around 0.68–0.73 [13].

Computer-based analysis has been attempted to overcome errors and increase the reliability and efficiency of diagnosis. Computerized reading has several advantages, including more objective and fast analysis, reduced cost of training high-level clinical specialists, and provision of a platform for large-scale screening [14]. The focus of early computer-assisted image processing technology was on facilitating readings by clinical specialists via enhancing the medical images in which subtle data differences could be distinguished; this was achieved by processing the values of pixels in MR images or automatically segmenting boundaries and objects using active shape models on X-ray images, such as video fluoroscopic images [15]. With the subsequent development of computer-based technologies, studies that attempted to automatically identify specific diseases using computer vision and machine learning (ML) technologies were introduced beyond simply assisting readings by clinicians. Early computer-based diagnostic studies employed feature extraction methods based on handcrafted features, such as scale-invariant feature transform (SIFT) [16], histogram of gradient (HOG) [17], or Harr-like feature [18]. However, these methods have shown limited performance for medical images with nonquantitative characteristics as well as atypical and complex features because they extract fixed or purpose-oriented features for specific tasks.

The recent rapid development of deep-learning techniques in ML has inspired increasing use of deep-learning-based image analyses for diagnosing specific diseases [19,20]. convolutional neural network (CNN) constitutes a type of artificial neural network that use convolutional operations [21]. Unlike traditional artificial neural networks that use vector-type inputs, the CNN can utilize spatial correlation information because it can accept two-dimensional data, such as images, as inputs. Since the introduction of CNN-based deep-learning networks such as AlexNet [21], various classification models, such as GoogleNet [22], VGGNet [23], ResNet [24], and EfficientNet [25], have been developed in addition to U-Net [26] for image segmentation and YOLO [27] or RetinaNet [28] for object detection; these are some examples for utilization and analysis of image data for diverse purposes. Deep-learning technology has achieved remarkable performance in general RGB image analyses, and various studies are actively using deep learning for medical image analyses.

For example, one study attempted to diagnose Alzheimer’s disease (AD) via analysis of brain MR images [29]; unlike existing methods in which features are manually extracted directly from MR images for use as inputs to the model [19], this method automatically extracts features related to AD by learning the 3D voxel patch type from landmarks set using a data-driven discovery algorithm [30]. Furthermore, additional data such as gender, age, and learning level were used to construct fully connected networks (FCNs) with CNN characteristics for the final diagnosis. Another study utilized positron emission tomography (PET) as well as MR images for AD diagnosis [31,32]. This approach extracted features from 3D volume MR and PET images using 3D convolution [33] for more effective extraction of semantic and spatial information through a bidirectional architecture with bidirectional long short-term memory (Bi-LSTM) [34]. In [35], a method for classifying three types of brain tumors using ResNet was proposed. In [36], a method was proposed to classify skin cancer using a CNN-based deep learning network, at a dermatological level. A single CNN [37] that was trained end-to-end using images was used, and pixels and disease labels were used as inputs. In [38], CNN was also used to automatically detect and localize tumors in gigapixel pathology images. Given the recent COVID-19 pandemic, a new technique has been developed to determine the presence of the COVID-19 infection based on a CNN analysis of chest medical images [39,40]. In [39], a mask was created to divide the lung area in chest CT images using a pre-trained U-Net, which was then used as the input to a 3D CNN with the original CT images to determine viral infection. Another study presented detection and scoring of cardiovascular disease from cardiac and chest CT images by automatic lesion slice assignment and scoring using a CNN regressor [41]. However, deep-learning-based image analysis studies focus mainly on disease groups that can be diagnosed using CT or X-ray images, for which a large number of databases are relatively easily secured; further, MR studies are focused mainly on brain MR imaging, MR angiography, or chest MR data, which are images of specific body parts.

Since information regarding lesions in the brain MR data is usually contained more in slices than in a 3D volume, many studies on brain MR imaging have applied CNN to slices. In contrast, chest MR imaging mainly uses a segmentation model for the input data in the form of a 3D volume of the chest to distinguish different organs rather than directly detecting any associated diseases [19]. Accordingly, various types of deep-learning methods have been utilized for MR image analyses depending on the purpose.

This study proposes a method for diagnosing bone marrow edema of the sacroiliac joint in patients with axSpA using deep-learning-based MR image analysis. This study was approved by the institutional review board of Chung-Ang University Hospital, Seoul, Korea (2011-002-402).

## 2. Materials and Methods

**Figure 1:**
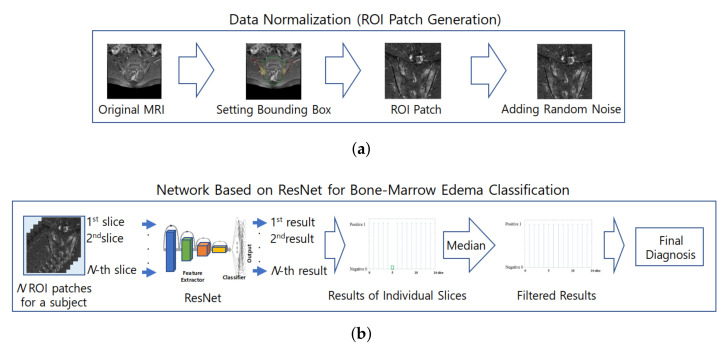
Overall process of the proposed method for diagnosing sacroiliac arthritis. (**a**) Process of ROI generation; (**b**) ResNet based network for Bone marrow edema classification.

The classification of subjects for the diagnosis of sacroiliac arthritis in this study largely consists of two parts. In the first part, the proposed method establishes a region of interest (ROI). ROI images are generated from the original MR images by removal of areas other than the sacral and iliac bones, where sacroiliac arthritis occurs; then, the ROI images are normalized for use in the deep-learning network. The second part involves determining the presence of bone marrow edema using a CNN-based deep-learning network on individual MR images and deciding whether a subject has sacroiliac arthritis based on the classification results of their individual MR images. Figure 1 shows the overall process for diagnosing sacroiliac arthritis as proposed in this paper.

### 2.1. Dataset

The MR images used in this study consist of a total of 815 slices of the SIJ region and were obtained from 79 subjects who visited Chung-Ang University Hospital. Of these, 60 subjects were diagnosed with axSpA by a rheumatologist according to the classification criteria [42] of the 2009 Assessment of SpondyloArthritis International Society (ASAS), and the remaining 19 subjects were classified as normal without sacroiliitis. The average age of the patients with axSpA and control were 32.3 ± 9.8 and 27.1 ± 11.4 years in the age ranges 18–59 and 18–55, respectively (*p* = 0.055). The male ratio was 71.7% (43/60) in axSpA population and 68.4% (13/19) in controls, respectively (*p* = 0.779). The MR images used in the study were obtained using a 3 T MR device (Skyra, Siemens Healthcare, Erlangen, Germany) as gadolinium-enhanced fat-suppressed T1-weighted oblique coronal images. 2D multi-slice Gadolinium-enhanced T1-weighted oblique coronal sequence was obtained approximately 10 min after a Gadolinium contrast injection. Detailed MR parameters were as follows: TR-544 ms, TE-11 ms, flip angle-140 degree, bandwidth-326 Hz, FOV-250 × 250 mm: slice thickness-2 mm, slice gap-0.2 mm: matrix-448 × 448, slice number-37, average scan time-3 min 14 s with fat-suppression using the DIXON method. All images were labeled under two classes (patient: positive, normal: negative)—bone marrow edema and normal—with 422 slices from the 60 axSpA subjects and 393 slices from the 19 normal subjects.

**Figure 2:**
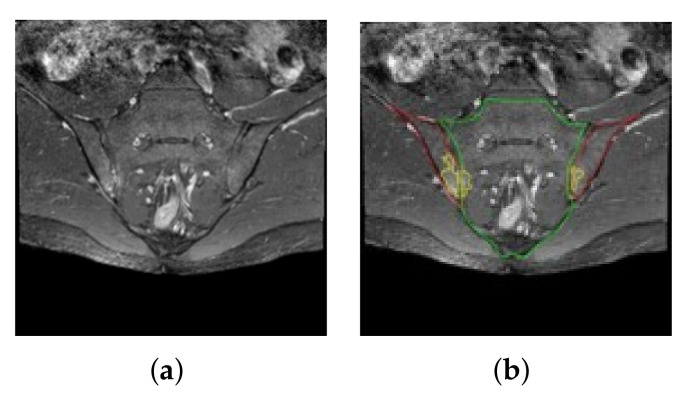
Example MR images of the SIJ region. (**a**) MR images including all areas surrounding the sacral and iliac bones; (**b**) annotated MR images of the sacral (green) and iliac (red) bones as well as bone marrow edema (yellow).

Figure 2 shows examples of the MR images of the SIJ region. As shown in Figure 2a, the images include all areas surrounding the sacral and iliac bones. Active sacroiliitis induces bone marrow edema in the joints between the sacral and iliac bones and was identified by checking for bone marrow edema on the MR images (area marked in yellow in Figure 2b). For deep learning of the images from subjects with axSpA, the boundary between the sacrum and ilium as well as the presence and extent of bone marrow edema were determined based on the results agreed upon by at least two members from among a team of two rheumatologists and one radiologist. The presence of active sacroiliitis was determined based on whether there were at least two bone marrow edema lesions on one image slice or whether bone marrow edema was found in at least two consecutive image slices [43]. Since lesions are not observed in all slices among the images of axSpA subjects, there may be problems in training the classification network for individual MR image slices. To eliminate this risk, only those slices in which lesions were clearly observed were used as the positive samples. Consequently, the MR images used in the study included a minimum of four to a maximum of 23 slices per axSpA subject, and a minimum of 18 to a maximum of 25 slices were used for each control subject.

### 2.2. Data Normalization for Deep-Learning Network

**Figure 3:**
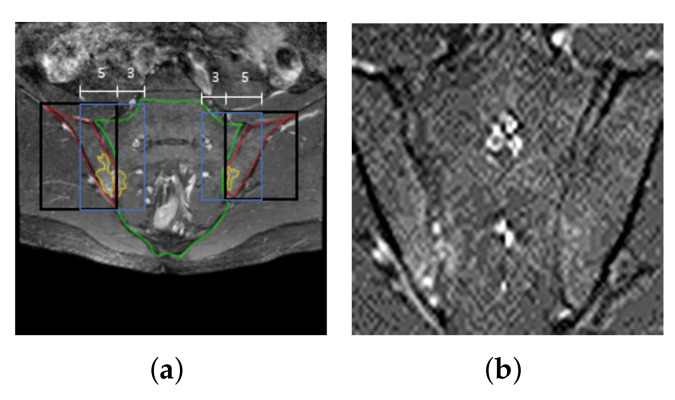
Data normalization. (**a**) Bounding boxes for the ROI (blue boxes) containing the left and right iliac bones (black boxes)); (**b**) ROI patch combining bounding boxes obtained from left and right iliac bone regions (blue boxes in (**a**)) with random noise.

MR images can contain local inhomogeneities in the Rician noise originating from inhomogeneities of the receiving sensors (coils) or from the image reconstruction, such as parallel imaging reconstruction. Therefore, we added random noise to compensate for the noise that may occur during MR image acquisition, and thus it was applied to all MR images taken for basic preprocessing, such as training, validation, and testing, regardless of the usage of the data [44,45]. To reproduce the texture of the MR images taken, we applied a Gaussian random variable $N_{G}\left(x,y\right)$ with zero mean and a variance of 0.3 as well as a Poisson random variable $N_{P}\left(X\right)$, where the log of a single pixel value extracted from an image and raised to the fourth power was set as the expected value, for all the MR images (Figure 3b).

The convolution-operation-based deep-learning network extracts spatial features from images by applying image filters to all regions of an image. This is because the regions other than the SIJs, which affect the diagnosis of active sacroiliitis, can induce operations unnecessary for deriving classification results and also act as interference for the extraction of features important for the diagnosis of sacroiliitis. Hence, in this study, the ROI was set as the areas around the sacral and iliac bones, annotated by a radiologist, in the MR image of the SIJs. From observations of the locations where bone marrow edema was determined in the positive samples, new ROI patches were created by additionally designating areas outside the bounding boxes in the direction of the sacral bone as well as half the area of the sacral bone side (green line) based on the centers of the bounding boxes containing the left and right iliac bones (red lines in Figure 3a). In other words, the new ROI includes the joints between the sacrum and ilium where the lesions are located. Consequently, the ROI corresponds to bounding boxes (blue boxes in Figure 3a) inward by as much as 0.8 times the width of the black bounding box. Patches are created one each on the left and right sides of the SIJs. If the heights of the left and right regions do not match, then height of the smaller patch was adjusted to that of the larger one based on the higher side.

### 2.3. Design of Deep-Learning Network for Diagnosing Sacroiliitis

The method proposed herein first determines presence of bone marrow edema based on the brightness distribution of the pixels in the SIJ region of each MR slice. In addition, the determination of whether a subject had active sacroiliitis was performed using contextual information based on the positional relationships between consecutive slices. It is relatively likely that when bone marrow edema is found in one slice, it may be present in adjacent slices as the MR slices from the lower to upper pelvis have a sequential positional relationship. Therefore, filtering [46] was performed in the direction of the one-dimensional spatial axis on the classification results of individual MR slices to detect the continuous presence of bone marrow edema in adjacent slices.

Among the MR images of the SIJ region, the ROI patches from around the sacrum and ilium were used as the input images to diagnose bone marrow edema. In gadolinium-enhanced fat-suppressed T1-weighted oblique coronal images, bone marrow edema caused by active sacroiliitis appears as bright pixels compared to the normal areas (Figure 2b).

**Figure 4:**
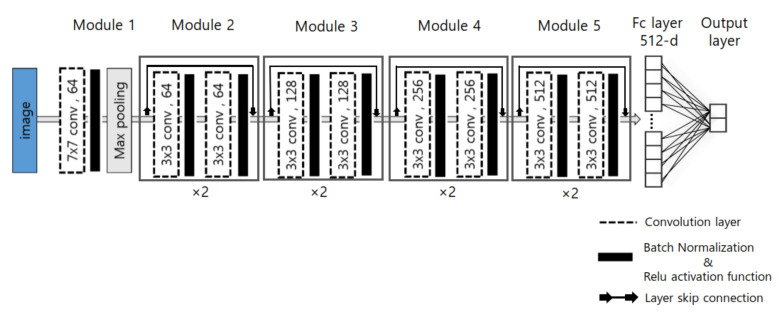
Configuration of ResNet consisting of five convolutional stages, which contain the convolution blocks comprising 7×7 and 3×3 layers. The output layer consists of as many nodes as the number of classes to be obtained.

The deep-learning network consists of numerous edges between neurons, and weights are assigned to each of these edges. The weights of the network are then trained using a back propagation algorithm [47], and the weight of each edge is updated using the derivative of the weight of the loss function. In many cases, the larger the depth, the better is the performance of the network in deep-learning networks [22,23,24,48]. In contrast, if the number of layers in the network increases, the “gradient vanishing’’ phenomenon [49,50] occurs where the weights of the layers close to the input layer are not accurately updated since the gradient becomes smaller toward the input layer in the back propagation process. To resolve this problem, ResNet [24] was used in this study to prevent gradient vanishing using skip connections to construct a deeper network more effectively. The ResNet consists of five convolutional stages (Figure 4), and the first convolutional stage contains a convolution block consisting of layers having 64 filters of size 7×7. From the second to fifth convolutional stages, the convolution blocks comprise 3×3 layers. From the third convolution stage, a convolution block in which 1×1, 3×3, and 1×1 layers are sequentially arranged was constructed instead of a 3×3 filter-based convolution block to prevent the number of parameters in the layer from increasing when designing the deep network. The output of the fifth convolution stage was then flattened to form a single feature vector, which is then used as the input to the fully connected layer. The fully connected layer consists of one hidden layer, excluding the output layer. The output layer consists of as many nodes as the number of classes to be obtained, and each node calculates the final output value by applying the sum of the weights of the nodes of the previous layer to a sigmoid function. For the diagnosis of active sacroiliitis, we defined the MR slices in which bone marrow edema was found as positive samples, and the remaining images were designated as negative samples. The design was aimed at generating a binary output.

The deep-learning network requires sufficient training data to avoid overfitting as the network depth increases. Because it is highly expensive to acquire large numbers of MR images for the diagnosis of active sacroiliitis, we used the transfer learning technique [51,52] to train the network effectively with a relatively small amount of training data. As noted in a previous study, the pre-trained ResNet [24] was applied as the backbone using ImageNet [53], which is a large image database commonly used in image recognition, by resizing the ROI patches to a size of 224×224.

The gradient-weighted class activation mapping (Grad-CAM) method [54] was used to analyze the reason for predicting the class in a deep-learning classification network using CNN. In this study, Grad-CAM was used to determine the area of the input image that primarily affected the final results so as to intuitively confirm the validity of the classification results of MR slices with bone marrow edema.

### 2.4. Evaluation Metric

The diagnostic performance for active sacroiliitis was evaluated based on (1) the ability to distinguish whether bone marrow edema was present in the individual MR slices using ResNet18-based CNN, and (2) the accuracy of whether a subject could be diagnosed with active sacroiliitis based on the results of presence of bone marrow edema in all MR slices from that subject.

The classification of MR slices with and without bone marrow edema at the SIJs is a type of one-class classification problem. Therefore, the performance of the proposed ResNet-based classification network was evaluated through the receiver operating characteristics (ROC) curve, which is mainly used in detection problems. Based on the predictive and ground truth values of the classification, the case where the predictive and ground truth values are identical is defined as true positive (TP) and true negative (TN), and the case where the predictive and ground truth values are different is defined as false positive (FP) and false negative (FN); the major metrics for performance evaluation are as follows: (1) Accuracy ((TP + TN)/(TP + TN + FN + FP)): the proportion of correctly classified samples with respect to all positive and negative samples; (2) Recall (TP/(TP + FN)): the proportion of samples that the classifier correctly determines as positive with respect to all actual positive samples; (3) Precision (TP/(TP + FP)): the proportion of samples that the classifier correctly determines as positive with respect to the number of samples that the classifier determines as positive; (4) Specificity (TN/(TN + FP)): the proportion of samples that the classifier correctly determines as negative with respect to the number of samples that the classifier determines as negative; (5) Negative prediction value (NPV): the proportion of samples that the classifier correctly determines as negative with respect to the number of samples that the classifier determines as negative; and (6) F1-score (2*(Precision*Recall)/(Precision + Recall)): harmonic average of recall and precision.

### 2.5. Network Training and Validation

For the experiments, a computer with the following specifications was used: Intel(r) core(tm) i5-7500, CPU@3.4 GHz, RAM 16 GB, NVIDIA Geforce GTX1070, Ubuntu 18.04 PyCharm. The framework used for analysis was PyTorch 0.4 CUDA 9.0 version.

Owing to the use of a nonlinear activation function in a deep-learning network, an internal covariance shift phenomenon [55] may occur where the distribution of the input data would change as it passes through the layers. To prevent this phenomenon and to increase the stability of training, batch normalization [55] was performed to adjust the mean and variance of the data during training. An appropriate batch size should be configured in deep learning that is sensitive to gradient values because the possibility of falling into a local minima is high if the batch is extremely large. Thus, the batch size was set to 32 based on the experimental results in this study. The learning rate was initially used to swiftly increase convergence speed, and the value was later lowered as the learning progressed using the cosine decaying method of Loshchilov [56] to ensure that the global minimum was effectively attained; the initial value was set to 0.03.

Typically, when training a deep-learning network, data is augmented using various methods, such as rotation, horizontal flipping, and jittering, to prevent overfitting and to reinforce robustness against spatial changes in the data. However, if “random crop’’ is applied among the augmentation techniques used in typical image classification, a positive subject can be misdiagnosed as normal because the location of the lesion that manifests in an atypical form is cut off. Thus, the data were augmented using only horizontal flipping and rotation in this study.

As the cost of MR examination is high, MR scans are often performed to confirm clinical signs of sacroiliitis. Thus, MR images of the SIJ regions tend to be collected more from positive subjects than normal (negative) subjects, as shown in Table 1. Accordingly, a dataset was constructed for this study to ensure that the ratio of positive and negative samples was similar. To alleviate the problem of unbalanced data volumes between classes, the focal loss function FL [28] was used instead of the cross-entropy loss, unlike general classification problems.

The primary goal of this study is to determine bone marrow edema based on MR images, but diagnosing active sacroiliitis in a specific subject is the ultimate aim. Thus, the training and test datasets were separated based on the subjects and not on individual MR slices. The training dataset was prepared by randomly selecting data from 70% of the axSpA and normal subjects, and the test dataset was prepared with data from the remaining 30%. To increase statistical reliability, the above operation was repeated five times to calculate the average classification rates of five-fold sets. Table 1 shows the composition information for the training and test data for each fold.

**Figure 5:**
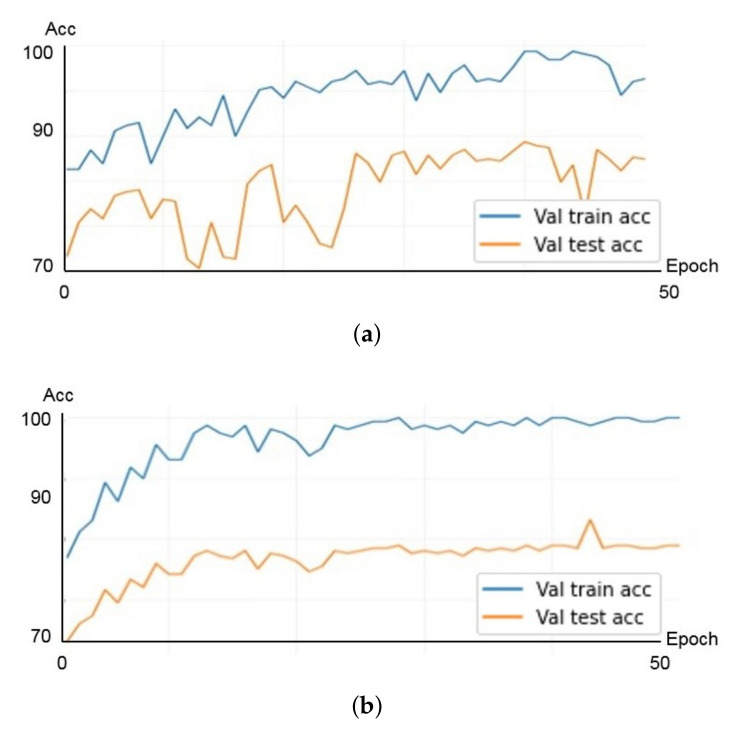
Results of validation experiment. (**a**) ResNet50 model; (**b**) ResNet18 model.

The ResNet architecture used in the proposed method has five types of models depending on the depth of the network. Experiments were performed on an 18-layer ResNet18 model and a 50-layer ResNet50 model to select a network with a more appropriate size for the data. Initially, about 30% of the training data of fold set 1 shown in Table 1 were separated and used as a validation set. Figure 5 shows the results of the validation experiment to check for overfitting of each model. In Figure 5, the horizontal axis represents the number of epochs of training, and the vertical axis represents the classification rate. The blue line corresponds to the results of the training data, and the red line corresponds to the results of the validation data. As shown in Figure 5, when the same dataset is used for both the ResNet18 and ResNet50 models, the ResNet18 model converges more stably for both the training and validation datasets for training up to 50 epochs. From these results, a classification network using the ResNet18 model was applied to the subsequent experiments.

## 3. Results

### 3.1. Performance for Sacroiliitis Diagnosis

**Figure 6:**
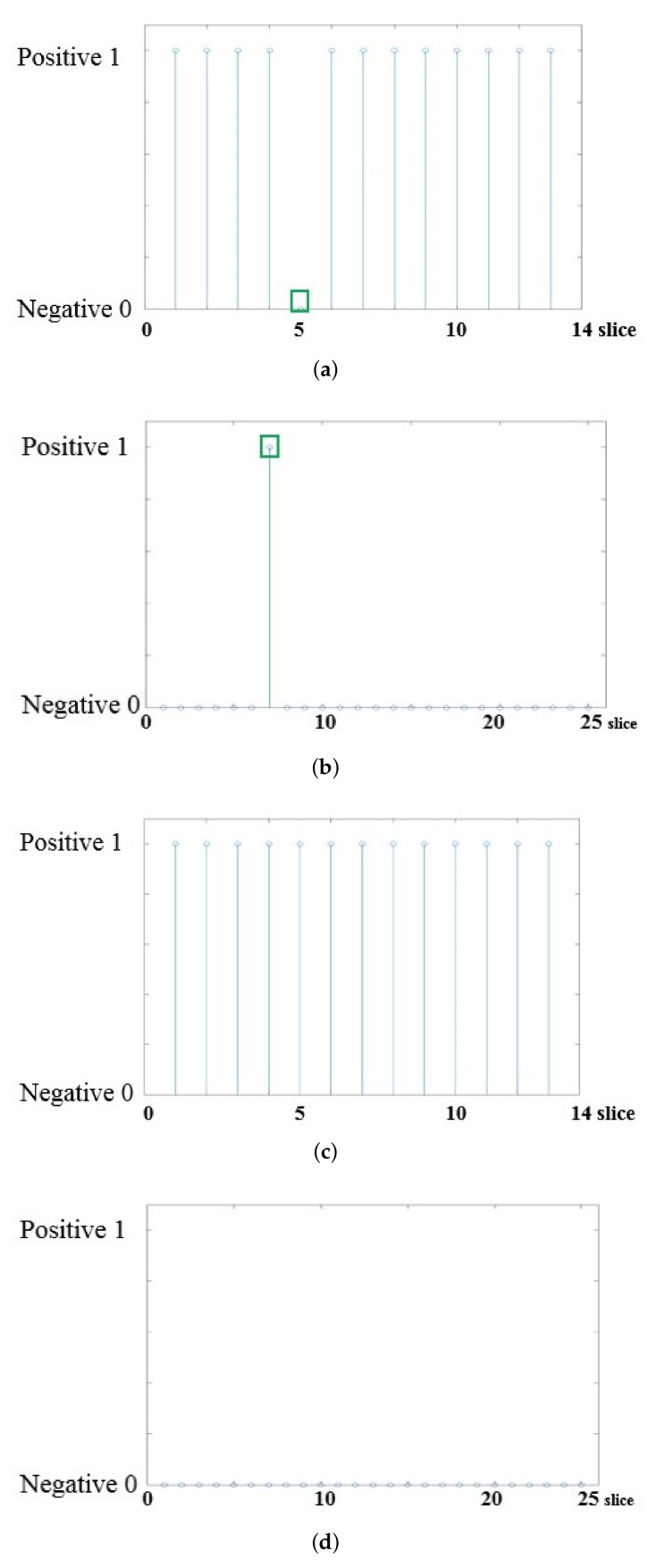
Results of automatically determining the presence of bone marrow edema using ResNet. (**a**) Results for axSpA subjects; (**b**) results for normal subjects; (**c**) results of applying the median filter to the results of (**a**); (**d**) results of applying the median filter to the results of (**b**).

Figure 6 shows the results of automatically determining the presence of bone-marrow edema using ResNet from MR slices of axSpA and normal subjects in this study (Figure 6a: results for axSpA subjects, Figure 6b: results for normal subjects). In Figure 6, the horizontal axis represents the index when the MR slices are sequentially arranged from the lower to upper pelvis, and the vertical axis represents the classification result of ResNet for each slice. When a slice is classified as positive, a value of 1 is indicated, and when classified as negative, a value of 0 is indicated. First, the results of all MR slices for a single subject were stored in the form of a vector, and the final diagnosis of active sacroiliitis was determined for the subject through one-dimensional spatial filtering [46]. Since the slices corresponding to the adjacent indexes are the results of scanning adjacent parts, as shown in Figure 6, it is relatively likely that bone marrow edema is simultaneously found in adjacent slices containing the corresponding areas if bone marrow edema occurs in one area of the SIJ. Thus, if bone marrow edema is intermittently detected, as shown in the green box in Figure 6a, or if bone marrow edema is not detected between other slices with detected bone marrow edema, as shown in the green box in Figure 6b, the results could be considered as misclassifications. Therefore, the samples that were misclassified in normal subjects (samples classified as positive) were corrected using a size-three median filter [46] to reflect the spatial sequential characteristics of the MR slices in the final diagnosis. Figure 6c,d show the results of applying the median filter to the results of Figure 6a,b, respectively. As shown in the figures, the false detection results can be corrected by filtering. The final diagnosis of sacroiliac arthritis for a single subject was then performed by averaging the results of the individual slices.

**Figure 7:**
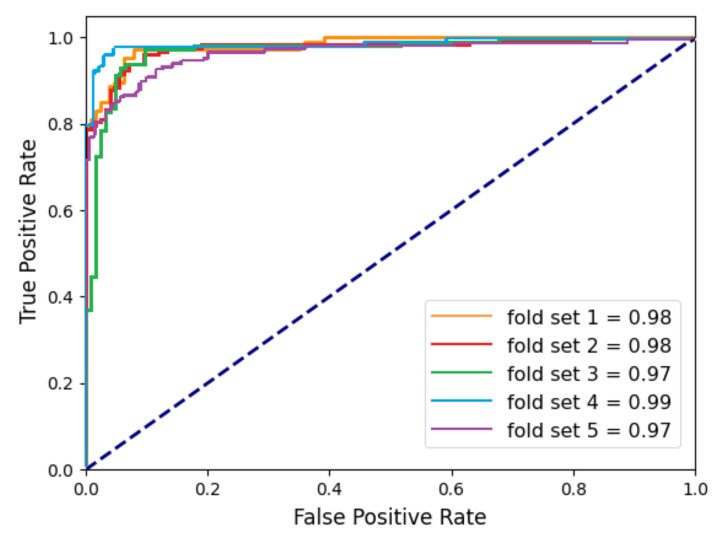
ROC curves for bone marrow edema classifications from individual MR slices.

To confirm the effects of the ROI patch setting described in Section 2.2, the results of the classification experiments were compared when the original MR slices were used in the deep-learning network (Figure 3a) and when the ROI patches were used (Figure 3b). Table 2 shows the performance of the ResNet18-based classification network for individual MR slices. As shown in Table 2, when the ROI patch was used, the accuracy was 93.55%, with 92.87% recall and 94.69% precision, indicating excellent detection performance of bone marrow edema. Compared to the case of using the original MR image, the case of using the ROI patch showed an average of 10.10%, 7.74%, and 12.79% higher performance for accuracy, recall, and precision, respectively (Figure 7). Regarding the standard deviation in performance based on the fold set, the case using the ROI patch showed smaller variations than the case using the original MR image. These results show that the use of the ROI patch helps the network focus more on the key areas for determining bone marrow edema by removing unnecessary information from the MR slices. Applying the median filter improved the overall performance slightly, as shown in Table 2. Figure 7 shows the ROC curves for classifying bone marrow edema for individual MR slices after applying the median filter. The area under the curve (AUC) of the ROC curves for bone marrow edema for each of the five-fold set were 0.98, 0.98, 0.97, 0.99, and 0.97.

Table 3 shows the final diagnosis for a subject, that is, the results of evaluating whether an individual has acute sacroiliitis based on the results of all slices of one patient. In Table 3, the final diagnosis of active sacroiliitis for each subject showed excellent performance, with 96.06% accuracy, 100% recall, and 94.84% precision.

### 3.2. Grad-Cam Result

**Figure 8:**
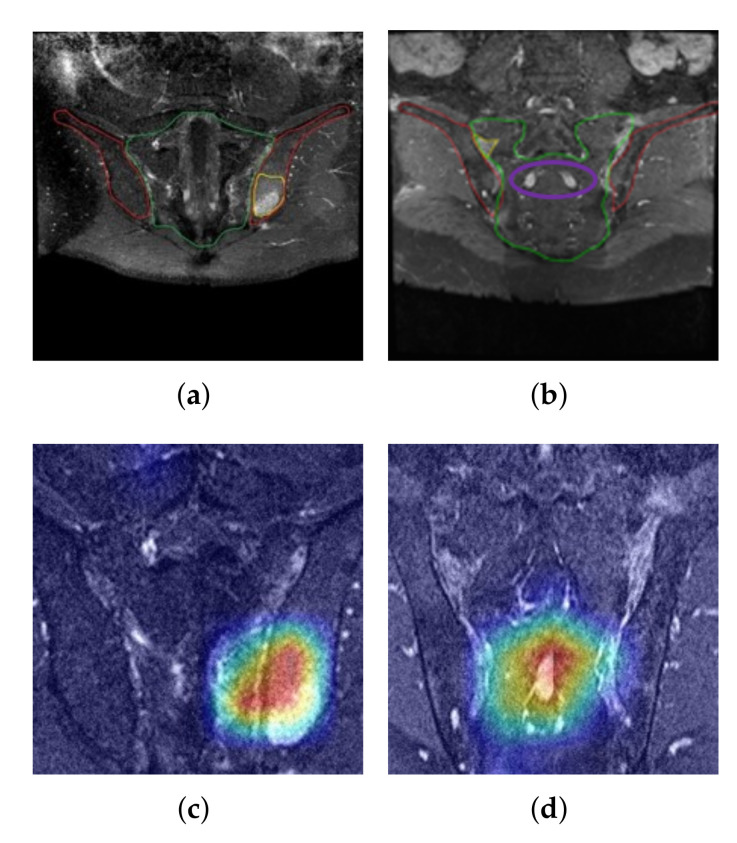
(**a**) Results of the class activation mapping for an example image with bone marrow edema; (**b**) Another example of an image with markers for bone marrow edema; (**c**) Gradient-based class activation map of (**a**); (**d**) Gradient-based class activation map of (**b**). The color represents regions with the greatest activation, and the degree of activation decreases in the order of orange, yellow, green, and blue.

Figure 8a,b are positive samples with bone marrow edema (areas marked in yellow), whereas Figure 8c,d are the results of extracting the class activation map for the positive class from the last convolutional layer of ResNet18 given the images of Figure 8a,b as inputs, respectively, and mapping onto the input images. The areas marked in red in Figure 8c,d represent regions with the greatest activation, and the degree of activation decreases in the order of orange, yellow, green, and blue. Figure 8a,c show that the area used by the proposed network to predict the sample as positive coincides considerably with the area containing the lesion. This result suggests that the process of positively determining the sample using the proposed method is reasonable based on the information of the actual lesion area in addition to the numerical results of the classification rate. Meanwhile, Figure 8b,d show that the area activated by the network for presence of bone marrow edema was concentrated around the sacral bone rather than the SIJ region. This is because the structural features of the sacral bone area (marked in purple), which are indicated by bright pixels in the corresponding MR slice in Figure 8b, interfered with the detection of the bright pixel groups owing to bone marrow edema Figure 8d. These errors could be one of the causes for the reduced reliability of deep learning networks. Therefore, these errors may be overcome in the future if more sophisticated segmentation of the SIJ area were to become available for MR slices.

## 4. Discussions and Conclusions

This study was aimed at developing a method to detect bone marrow edema from MR images of axSpA subjects using deep-learning techniques. The ResNet-based network designed in this study achieved 93.80% accuracy, 93.35% recall, and 94.70% precision for detecting active sacroiliitis based on regions exhibiting bone marrow edema in MR images. Considering that the concordance rate between specialists on the occurrence of sacroiliitis for diagnosing axSpA is not high [8,10,13], our results suggest that MR analysis using deep learning could be useful as an auxiliary means of diagnosing active sacroiliitis.

This study has great significance from the viewpoint of being a pioneering work that uses deep learning to diagnose bone marrow edema in subjects with axSpA. With regard to the medical domain, it is challenging to simply apply the methods used in other fields because there are marked differences depending on the research direction and approach according to the characteristics of the organs or diseases. For acute sacroiliitis, the target disease of this study, the amount of MR imaging data is relatively small compared to other diseases. There are no reported studies on application of deep learning to the SIJ. This study is a first attempt and presents the design of a deep-learning network that can effectively diagnose bone marrow edema using transfer learning, with only a small number of MR images of bone marrow edema.

The method used in this study has the advantage that it does not require MR data in the form of a complete volume, and analysis can be conducted only with MR images in the form of a ROI patch, unlike conventional methods. Thus, we confirm that performance improvement is possible by simply creating ROI patches from images.

Owing to the differences in signal intensities in individual images, it is essential to correct for differences in the brightness of the images for comparison and detection of bone marrow edema in deep learning. In several deep learning studies, random noise is often added to the data to create a noise-resistant network [44]. To this end, we added random noise to the input images to alleviate the image-shift problem based on the type of equipment used or differences in the imaging environment.

This study uses a method of classifying patients via deep learning by adopting patch-wise inputs to the reconstructed SIJ MR data based on the findings of specialists and by presenting the basis of the results using Grad-CAM. The results show that lesions can be discovered via Grad-CAM without separate instructions from the model, which is one of the advantages of the proposed method compared to conventional classification models that cannot provide the necessary reasoning for diagnosis. However, there are still instances where parts of the image with high MR signal intensities in regions other than the SIJs were erroneously detected as bone marrow edema, as shown in Figure 8d. This may be one of the factors producing FPs in this work. If sophisticated segmentation processing for the SIJ area becomes available through further study, the rate of FPs may decrease.

It is necessary to first identify the sacrum and ilium in the MR images to find the SIJ. The proposed network could also perform a fully automatic classification of bone marrow edema using whole MR images as input without ROI setting. In Table 2, the result for ’Ori data’ is a fully automatic classification of bone marrow edema without manual ROI setting. As in Table 2, it also showed a high accuracy of 83.45% for ‘Ori data’. The result of the second row in Table 2 is the classification rates when using the ROI patch for each MRI slice, which corresponds to Figure 5a,c. The comparison of the first row and the second row in Table 2 shows the effect of using the ROI patch. The classification performance was further improved by 93.80% when the ROI was set up because the network could focus more on the region where lesions occur. Because the network classification rates for each MRI slice is not perfect, the results (the third row in Table 2) of each slice were corrected using a median filter to utilize the spatial sequential characteristics of MRI slices. If bone marrow edema occurs in one part of the joint, all the consecutive slices in that area are likely to be classified as positive. Therefore, if one of the three consecutive slices is classified as negative, the result is likely to be an error. Median filter is a useful filter for correcting these outlier samples. In Table 2, the value of ’ROI patch (median filter)’ indicates the application of the processes in Figure 5b,c.

Owing to the difficulties of learning such as overfitting when using 3D-based deep learning techniques for small data, in this study, we designed a 2D-based network that can utilize ‘pre-training’ and ‘transfer learning’. In several cases, the transfer learning we employed is effective for applying deep learning techniques to small datasets, but not for all datasets. Depending on the data property, there exist studies that have used ResNet by applying transfer learning to small datasets with fewer than 1000 samples [57,58]. If more data is available in the future, we will continue to investigate the applicability of a 3D CNN model that can utilize the spatial positional relationship information between MR slices in the network learning process.

These bones have atypical shapes unlike the square-shaped vertebral body; specifically, the SIJ has a more complex structure than other joint surfaces because it is widely distributed in a diagonal orientation. Thus, this study primarily required specialists to manually identify the boundaries between the sacrum and ilium. It is necessary to perform classification fully automatically from the ROI setting to the final decision to increase the effectiveness of the proposed method. However, in medical images, there are many non-rigid objects, and even the same object has large variations in shape depending on individuals, making it difficult to apply segmentation techniques for ordinary images like they are applied to medical images, and thus, further studies are required to distinguish the boundaries between the sacrum and ilium automatically. Furthermore, through the designing of a spatial context-based network that simultaneously uses multiple sequential MR images as inputs, or via the use of a 3D-based deep learning model, the median filtering step that follows classification for individual MR images can be merged into one network with the classification network to simplify the overall structure. We are conducting research on a semantic segmentation method that can be applied to MR images for an automatic ROI setting, as a follow-up study.

This study was focused only on the diagnosis of active sacroiliitis, the application of machine learning to the quantification of edema over time, or under treatment could be clinically very useful in various ways as well; hence, we intend to conduct further investigations in the future by comparing with clinical indices, such as ankylosing spondylitis disease activity score, bath ankylosing spondylitis disease activity index, and the spondyloarthritis research consortium of Canada, to determine whether the proposed method can appropriately reflect disease progression.

This study defined active sacroiliitis in subjects with axSpA as bone marrow edema. The main characteristic of active sacroiliitis in axSpA is bone marrow edema, and the ASAS/OMERACT consensus defines active sacroiliitis as bone marrow edema alone [11]. Bone marrow edema is a characteristic that is used to diagnose a disease and to determine its progression [43,59]. Moreover, considering that active sacroiliitis can manifest in patients with axSpA as well as those with peripheral SpA [60], the method proposed herein may also be helpful for detecting inflammation of the SIJs in diseases other than axSpA. However, other lesions, such as synovitis, enthesitis, and capsulitis, may also be detected [61]. One of the primary limitations of this work was that the detection of such lesions was excluded. Furthermore, cases in which only joint deformities, such as bony erosion or bony ankylosis, remained without active sacroiliitis were also excluded. A contrast agent is often utilized for adjacent abscess identification or hypervascularization owing to osteitis, which could be one of the factors causing false positives. In particular, MR images have various types of settings, including T1-weighed images, T2-weighted images, and short-tau inversion recovery images; therefore, the types of lesions that can be detected may vary depending on the settings individually or in combination. Hence, to identify lesions that may appear in the SIJs of patients with axSpA as well as bone marrow edema in the future, it is necessary to investigate MR images with other settings in addition to the gadolinium-enhanced fat-suppressed T1-weighted images used in this study. Conversely, bone marrow edema of the sacroiliac joint can also occur as part of diseases other than axSpA; however, in this study, machine learning was performed only on patients confirmed by a rheumatologist to have active sacroiliitis, and patients with other diseases were excluded from the study. Therefore, it would be difficult to differentiate other diseases using the results of this study, which is an aspect that requires additional research. In addition, the clinical usefulness of this study needs to be evaluated through follow-up studies by comparing the diagnosis results of specialists with and without assistance from the method presented herein.

In conclusion, a method to extract the clinical imaging characteristics of bone-marrow edema is developed in this work by applying a deep-learning algorithm based on CNN to SIJ MR images; further, it is shown here that active sacroiliitis can be effectively identified using the proposed method. These results suggest that SIJ MR analysis using deep learning might be useful and applicable for diagnosing bone marrow edema.

## Figures and Tables

**Table 1:** Information on the compositions of the training and test data for each fold.

Fold Set	Train Dataset		Test Dataset	
	Positive	Negative	Positive	Negative
1	288 (41 people)	265 (13 people)	134 (19 people)	128 (6 people)
2	283 (41 people)	266 (13 people)	139 (19 people)	127 (6 people)
3	298 (39 people)	269 (13 people)	124 (21 people)	124 (6 people)
4	298 (40 people)	267 (13 people)	124 (20 people)	126 (6 people)
5	262 (41 people)	274 (13 people)	160 (19 people)	119 (6 people)

**Table 2:** Performance for classifying bone marrow edema from individual MR slices (%).

	Accuracy	Recall	Precision	Specificity	NPV	F1-Score
Ori data	83.45	85.13	81.90	82.72	87.03	82.68
	(±3.37)	(±8.26)	(±7.56)	(±7.08)	(±5.59)	(±4.92)
ROI patch	93.55	92.87	94.69	94.23	92.40	93.75
	(±2.19)	(±1.27)	(±3.03)	(±3.77)	(±2.31)	(±1.78)
ROI patch	93.80	93.35	94.70	94.24	92.87	93.95
(median filter)	(±2.38)	(±1.83)	(±3.01)	(±3.79)	(±2.68)	(±1.91)

**Table 3:** Performance for final diagnosis of active sacroiliitis for each subject (%).

Accuracy	Recall	Precision	Specificity	NPV	F1-Score
96.06	100	94.84	86.43	100	97.32
(±2.83)	(±0.00)	(±3.73)	(±8.89)	(±0.00)	(±1.97)

## Data Availability

Not available.
